# Empowered healing: Boosting confidence and resilience through self-affirmation in post-cesarean mothers

**DOI:** 10.6026/973206300212657

**Published:** 2025-08-31

**Authors:** Atchaya Rajagopal, Sumathi Chandran, Shankar Shanmugam Rajendran, Vanitha Narayanasamy Naidu, Azhgar Jan Thoulath Khan, Jessy Raja, Amudha Chinnappan

**Affiliations:** 1 Department of Obstetrics and Gynecological Nursing, College of Nursing, Madras Medical College, The Tamil Nadu Dr.MGR Medical University Chennai, Tamil Nadu, India; 2 Department of Obstetrics and Gynecology, Government Hospital for Women and Children, The Tamil Nadu Dr.MGR Medical University, Chennai, Tamil Nadu, India; 3 Department of Child Health Nursing, College of Nursing, Madras Medical College, The Tamil Nadu Dr.MGR Medical University, Chennai, Tamil Nadu, India; 4 Department of Community Health Nursing, College of Nursing, Madras Medical College, The Tamil Nadu Dr. MGR Medical University, Chennai, Tamil Nadu, India

**Keywords:** Positive self-affirmation, self-confidence, self-resilience, cesarean mothers

## Abstract

The impact of self-affirmation as a therapeutic intervention for postpartum mothers who have undergone cesarean sections, aiming to enhance their self-confidence and resilience is of interest. A true experimental design with pre-test and post-test measures was used, involving 60 mothers in both experimental and control groups. The results showed that the experimental group exhibited significant improvements in self-confidence and resilience following the intervention. Positive self-affirmation techniques, including educational materials on diet, hygiene, and newborn care, contributed to these improvements. Thus, we show that positive self-affirmation significantly enhanced psychological well-being and coping abilities among mothers who had cesarean sections.

## Background:

According to the self-affirmation theory, people are driven to maintain a positive self-perception and to counter threats to their perceived competence [1]. When someone feels threatened, self-affirmations can help them regain their sense of self-competence by enabling them to reflect on their values and other sources of self-worth [2]. However, there are still many unanswered questions about how self-affirmation works. An area of interest analysis revealed that when pondering future-oriented core values (as opposed to daily activities), affirmed individuals (compared to unaffirmed participants) displayed greater activity in key brain regions connected to self-processing and valuation [3]. The term "maternal self-confidence" refers to a mother's confidence in her ability to care for and understand her child. This confidence plays a critical role in accepting the obligations of motherhood, which is important for nurturing and developing newborns as well as improving newborn outcomes [4]. Self-Resilience is said to be based on experiences during important stages of physical and personal development as well as genetics. Resilience is a dynamic process that can be developed and improved over time rather than a permanent attribute [5]. A person's life circumstances play a significant role in shaping resilience, a trait that can develop and evolve over time. The level of resilience varies and is affected by factors such as age, gender, background, and personal experiences [6]. Individuals who are resilient tend to demonstrate enhanced social abilities, particularly in communication, building close relationships and exhibiting empathy, largely shaped by their family experiences. Therefore, it is of interest to demonstrate the positive impact of self-affirmation in enhancing self-confidence and resilience among postpartum mothers who have undergone cesarean sections. It offers valuable insights into improving psychological well-being and coping abilities in this specific population.

## Methodology:

A True Experimental study Design was used to assess effectiveness of positive self-affirmation on self-confidence and self-resilience among post cesarean mothers conducted at the Selected Tertiary care Hospital, Chennai, over four weeks. The study Population comprised those who met the inclusion criteria, including Primi post cesarean mothers and Primi cesarean Mothers who are willing to participate. Mothers who are sick during the study period and Mothers who are under high risk were excluded. A Total of 60 Mothers was included in the study. Participants were selected using a simple Random Sampling Technique to ensure feasibility and timely Data Collection. Tools used were LIPS Maternal self-confidence scale, Brief Resilience scale and the ethical principles were followed accordingly. The data was tabulated and analyzed using both descriptive and inferential statistics. In the study, a true experimental design was used to assess the effectiveness of positive self-affirmation on self-confidence and self-resilience among post-cesarean mothers. The data was analyzed using both descriptive and inferential statistics. In Table 1 (see PDF), the comparison of self-confidence scores between the experimental and control groups revealed a statistically significant difference in post-test scores. The experimental group showed a mean score of 36.93 (SD = 4.66), while the control group had a mean of 21.63 (SD = 3.7), with a mean difference of 15.30 (t = 16.02, p = 0.001***). This difference was highly significant. Similarly, Table 2 (see PDF) shows the comparison of self-resilience scores between the experimental and control groups. The experimental group had a post-test mean of 24.37 (SD = 1.8), and the control group had a post-test mean of 17.50 (SD = 2.8), with a mean difference of 6.87 (t = 11.39, p = 0.001***). This difference was also statistically significant. Figure 1 (see PDF) depicts the post-test level of self-resilience scores, while Figure 2 (see PDF) illustrates the post-test level of self-confidence scores.

## Results and Discussion:

In the pretest, 76.67% of the experimental group had negative scores, while 73.33% of the control group had negative scores. Both groups had no positive scores. These findings align with Escobar-Soler *et al.* (2023) [7], who found a significant difference in resilience levels before and after self-affirmation (p ≤ 0.005), with the control group showing no significant change (p ≥ 0.005). The experimental group showed a 33.33% gain in self-confidence, compared to 0.83% in the control group, indicating the effectiveness of positive self-affirmation. Similarly, the experimental group had a 26.33% gain in self-resilience, while the control group showed a 2.33% gain. These results support the findings of Hennessy *et al.* (2018) [8], who observed a significant improvement in confidence levels after affirmations (p = 0.00). Post-test, the experimental group had a mean self-confidence score increase of 16.00 points (from 20.93 to 36.93), which was statistically significant, while the control group had a small, non-significant increase of 0.40 points (from 21.23 to 21.63). Additionally, recent studies have explored the relationship between self-affirmation and psychological well-being, with Hajure *et al.* (2024) finding significant improvements in self-affirmation techniques on mental health outcomes [9]. Thitipitchayanant *et al.* (2018) also support the importance of such interventions in enhancing resilience and psychological well-being among various populations [10]. Finally, Jose *et al.* (2025) further highlighted the impact of psychological interventions on post-cesarean mothers, demonstrating the relevance of these findings in clinical settings [11].

## Conclusion:

The positive self-affirmation significantly improves self-confidence and resilience in cesarean mothers. By incorporating self-affirmation into postpartum care, mothers experience enhanced psychological well-being and better cope with recovery challenges. Thus, we show the importance of integrating emotional health strategies into standard postnatal care practices.

## References

[1] Cascio CN (2016). Soc Cogn Affect Neurosci..

[2] Dutcher JM (2016). Psychol Sci..

[3] Falk EB (2015). Proc Natl Acad Sci U S A..

[4] Tognasso G (2022). Int J Environ Res Public Health..

[5] Luthar SS (2000). Dev Psychopathol..

[6] Schetter CD (2011). Soc Personal Psychol Compass..

[7] Escobar-Soler C (2023). Healthcare (Basel)..

[8] Hennessy EA (2018). Campbell Syst Rev..

[9] Hajure M (2024). Front Psychiatry..

[10] Thitipitchayanant K (2018). Pak J Med Sci..

[11] Jose T (2025). Cureus..

